# Skilful multi-year predictions of tropical trans-basin climate variability

**DOI:** 10.1038/ncomms7869

**Published:** 2015-04-21

**Authors:** Yoshimitsu Chikamoto, Axel Timmermann, Jing-Jia Luo, Takashi Mochizuki, Masahide Kimoto, Masahiro Watanabe, Masayoshi Ishii, Shang-Ping Xie, Fei-Fei Jin

**Affiliations:** 1 International Pacific Research Center, University of Hawaii at Manoa, 1680 East-West Road, Honolulu, Hawaii 96822, USA; 2 Department of Oceanography, University of Hawaii at Manoa, 1680 East-West Road, Honolulu, Hawaii 96822, USA; 3 Centre for Australian Weather and Climate Research, Bureau of Meteorology, GPO Box 1289, Melbourne, Victoria 3001, Australia; 4 Japan Agency for Marine-Earth Science and Technology, 3173-25 Showa-machi, Kanazawa-ku, Yokohama, Kanagawa 236-0001, Japan; 5 Atmosphere and Ocean Research Institute, University of Tokyo, 5-1-5 Kashiwanoha, Kashiwa, Chiba 277-8568, Japan; 6 Meteorological Research Institute, Japan Meteorological Agency, 1-1 Nagamine, Tsukuba, Ibaraki 305-0052, Japan; 7 Scripps Institution of Oceanography, University of California San Diego, 9500 Gilman Drive MC 206, La Jolla, California 92093-0206, USA; 8 Department of Meteorology, University of Hawaii at Manoa, 2525 Correa Road, Honolulu, Hawaii 96822, USA

## Abstract

Tropical Pacific sea surface temperature anomalies influence the atmospheric circulation, impacting climate far beyond the tropics. The predictability of the corresponding atmospheric signals is typically limited to less than 1 year lead time. Here we present observational and modelling evidence for multi-year predictability of coherent trans-basin climate variations that are characterized by a zonal seesaw in tropical sea surface temperature and sea-level pressure between the Pacific and the other two ocean basins. State-of-the-art climate model forecasts initialized from a realistic ocean state show that the low-frequency trans-basin climate variability, which explains part of the El Niño Southern Oscillation flavours, can be predicted up to 3 years ahead, thus exceeding the predictive skill of current tropical climate forecasts for natural variability. This low-frequency variability emerges from the synchronization of ocean anomalies in all basins via global reorganizations of the atmospheric Walker Circulation.

## Abstract (web summary)

Sea surface temperature anomalies in the tropical Pacific can influence global atmospheric circulation, yet prediction of this atmospheric signal is limited to less than 1 year. Here, the authors present observational and modelling evidence for multi-year predictability.

## Graphical abstract

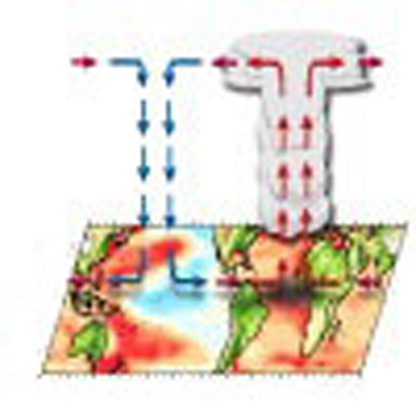

The Pacific El Niño Southern Oscillation (ENSO) is a major driver for interannual atmospheric and oceanic variability, which is well documented as a global teleconnection pattern^1^,^2^. On decadal timescales, tropical climate anomalies can affect global warming rate^3^, drought patterns^4^,^5^, regional sea-level changes^6^,^7^, the frequency and characteristics of El Niño events^8^ and the abundance of phytoplankton and movement of fish in the oceans^9^. As an example, the recent ∼20-year-cooling trend in the eastern tropical Pacific has been linked to the generation of massive droughts in Southern California^3^, an intensification of Pacific trade winds and accelerated sea-level rise in the western tropical Pacific^6^. This decadal-scale cooling has also contributed to the recent hiatus of global mean surface temperature rise^3^,^10^,^11^,^12^. Forecasting the phase reversal of the recent Pacific sea surface temperature (SST) trend several years ahead—beyond the typical ENSO prediction limit of ∼1 year^13^—would be of great societal benefit for instance for western Pacific island nations, which experience frequent inundations, and for the farming and energy sectors worldwide.

Climate predictions may exhibit enhanced skill on timescales of years to decades as a result of internal climate dynamics, or in response to slowly evolving boundary conditions associated with anthropogenic and natural radiative forcings, or combinations thereof. Although there is an emerging consensus that internally generated low-frequency climate variability in the extratropical North Atlantic and North Pacific^14^,^15^,^16^ may be predictable up to half a decade^17^,^18^, such skill is still elusive for the tropical climate system, where longer-term predictability seems to be mainly determined by external boundary forcing associated with changes in greenhouse gas (GHG)^19^ and aerosol concentrations^20^.

Here we revisit the subject of multi-year predictability of tropical Pacific climate, by focusing on the low-frequency modulation of trans-basin gradients in tropical SST and sea-level pressure (SLP) across the Atlantic/Indian/Pacific basins. Our study is based on a series of Coupled General Circulation Model hindcasts with the MIROC model^21^ that were initialized between 1960 and 2005 from realistic assimilated ocean conditions^16^ (see Methods). Every 5 years, a 10-year hindcast was performed and the time evolution of historical external forcings was prescribed during the forecast (see Methods). In addition, a second suite of uninitialized model experiments was conducted to investigate the contribution of ocean initial conditions to the multi-year predictability of the tropical climate. To evaluate the impact of ocean variability in the equatorial Pacific, the Atlantic and the Indian Ocean on multi-year predictability elsewhere, we also conducted a set of experiments for which observed ocean data were assimilated only in one of the ocean basins, whereas the other basins were allowed to evolve freely (see Methods). From these experiments, we show the multi-year predictive skills for the low-frequency tropical climate variability via global reorganizations of the atmospheric Walker Circulation.

## Results

### Predictability of tropical low-frequency climate variability

**Figure 1:**
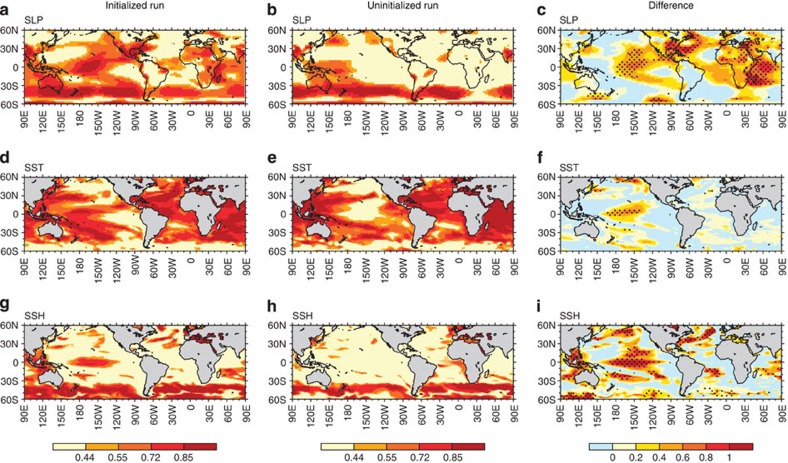
Predictive skills for 2–5 years lead time. Potential predictive skills of (**a**–**c**) SLP, (**d**–**f**) SST and (**g**–**i**) SSH anomalies for averaged 2–5 years lead time in the initialized run (left), the uninitialized run (center) and their difference (right). Predictive skills in the initialized hindcast and uninitialized runs are measured per grid-point by the anomaly correlation coefficient and with the same number of initial cases (that is, 10 cases) by comparing with the assimilation run that is comparable to the observation. Correlation coefficients of 0.44, 0.55, 0.72 and 0.85 correspond to the statistical significance at 90, 95, 99 and 99.9% levels with eight degrees of freedom on the basis of one-side Student's *t*-test. Dotted areas indicate the statistically significant (>90%) difference in the anomaly correlation skill between the initialized and the uninitialized runs, as measured by Fishers *z*-score.

The model forecasts demonstrate that the external radiative forcing is still the dominant source for multi-year SST predictability over major parts of the tropical oceans (Fig. 1). However, in the central Pacific, the situation is fundamentally different: the contribution of external forcing is almost negligible, particularly for SLP anomalies. In fact, most of the multi-year predictability of SST and SLP anomalies there results from the ocean initial conditions and hence the long-term memory of the ocean state (Fig. 1c,f). Consistent with these SLP and SST predictive skills, sea surface height (SSH) also shows the multi-year predictability in the central tropical Pacific and Maritime continent (Fig. 1g). This enhanced multi-year predictability in central Pacific SLP can be explained by the fact that SLP, and in particular wind variability, are more sensitive to variations in the zonal gradient of SST rather than to the nearly homogeneous SST warming^22^ induced by GHGs increases. We find that, unlike in the case of SST, natural variability explains >80% of the total variance of the SLP anomalies over major parts of the tropics (Supplementary Fig. 1). In the central tropical Pacific, therefore, the natural atmosphere–ocean variability is the major contributor for multi-year predictability.

In the Atlantic and Indian basins, we also find that the initialized run outperforms the SLP predictive skill of the uninitialized run over the tropical eastern Atlantic and western Indian Ocean, as well as the northwestern Atlantic (Fig. 1c). In the initialized run, SSH exhibits the multi-year predictability in the North Atlantic and near Madagascar (Fig. 1g). On the other hand, SST predictive skills are almost comparable between the initialized and uninitialized runs because of the dominant contribution from the externally forced component (Fig. 1d–f). This spatially organized ocean/atmosphere trans-basin variability, which involves unforced and forced heat content variability in different ocean basins, is synchronized by tropical atmosphere circulation changes as shown below.

### Trans-basin variability in the tropics

**Figure 2:**
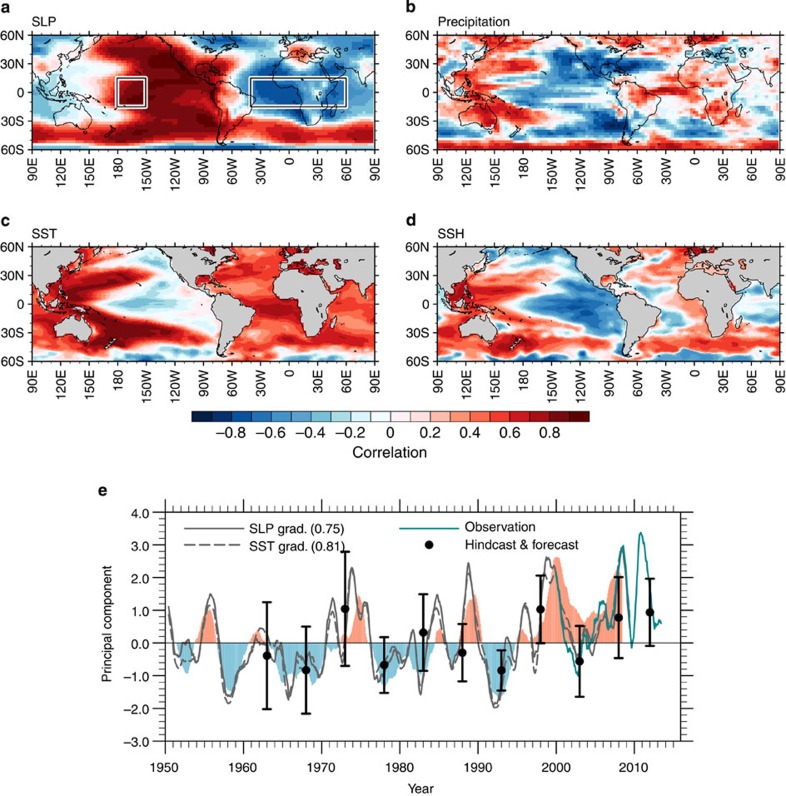
Patterns associated with the trans-basin variability. Correlation maps of (**a**) SLP, (**b**) precipitation, (**c**) SST and (**d**) SSH anomalies with the principal component (shaded in **e**) of the leading correlation-matrix based EOF for global SLP anomalies (60° S–60° N) smoothed by 36-month running mean filter (explaining 31.7% of variance) in the assimilation run. Grey and turquoise solid lines (**e**) denote the 12-month running mean tropical SLP-based TBV index (Pacific minus Atlantic SLP anomalies averaged over the boxes in **a** in the assimilation run and the observation (ERA-interim^43^) from 2000 to 2013, respectively. Broken line (**e**) is the tropical SST-based TBV index (bracket; correlation coefficient with the principal component). Black circles and bars denote the hindcasting and forecasting ensemble mean of the tropical SLP-based TBV index and its spread for 2–5 years lead time.

By determining the dominant mode of low-frequency SLP variability, we identify a pressure seesaw between the central tropical Pacific and Atlantic–Indian Ocean basins as well as its related patterns in SST, SSH and precipitation (Fig. 2). These patterns show the zonal gradients of SLP and SST anomalies between the central Pacific and tropical Atlantic/Indian Oceans. Moreover, precipitation anomalies exhibit the tropical Pacific–Atlantic contrast that is typical of a trans-basin displacement of Walker Circulation through atmosphere–ocean interactions. The trans-basin SST pattern is similar to that generated by climate models in response to specified Indian Ocean or Atlantic warming trends^5^,^23^,^24^,^25^. A large negative Atlantic/Pacific SLP gradient generates anomalous surface easterly (westerly) winds in the western Pacific (tropical Atlantic) and causes a zonal displacement of SSH anomalies in the tropical Pacific (Atlantic). We also find similar results of trans-basin displacements in the detrended anomalies or other observational re-analysis data sets for the different periods (Supplementary Figs 2 and 3). This coherent tropical low-frequency variability across the basins is referred to as tropical trans-basin variability (TBV)^5^ and represented by a tropical TBV index as the difference of standardized SLP anomalies in the tropical central Pacific minus the tropical Atlantic–Indian Oceans (see Methods).

Although TBV has a similar pattern to ENSO^26^,^27^ in the Pacific, it has very different characteristics in the Indian and Atlantic Oceans. In fact, SST anomalies in these areas oppose those of the typical ENSO teleconnection pattern^1^,^28^,^29^. The most discernable characteristic of the TBV, however, is the active role of ocean variability in the Atlantic. Obviously, SST variability in the equatorial Pacific can affect climate variability in the other oceans on interannual-to-decadal timescales^1^,^2^,^3^. In fact, we can capture a large fraction of tropical SLP variability^30^ by partially assimilating the ocean state in the equatorial Pacific only (Supplementary Fig. 4a and see Methods). Surprisingly, however, when we partially assimilate the Atlantic Ocean state, SLP variability in the central tropical Pacific is also captured well (local maximum of correlation coefficient in Supplementary Fig. 4c is 0.6; above statistical significance at 99.9% level). This Pacific SLP multi-year variability is thus partly generated by the Atlantic SST anomalies rather than by the external radiative forcings (Supplementary Fig. 4c,g). Associated with this Atlantic-induced Pacific SLP variability, wind-driven SSH variability also shows a positive correlation in the equatorial Pacific (Supplementary Fig. 4d). The central Pacific is one of the most predictable areas for SLP on multi-year timescales demonstrated by the initialized run (Fig. 1). According to these results, the long-term memory of the TBV and the associated multi-year predictability partly originate from the Atlantic Ocean. Despite the fact that the coupled climate model still has large biases to simulate the observed variability, the atmosphere–ocean anomalies associated with the TBV, in particular the low-frequency component, are captured well by the partial assimilation over the Atlantic Ocean (Supplementary Fig. 5). Compared with the Atlantic, the Indian Ocean plays a lesser role in the central Pacific SLP variability but still contributes more than the externally forced component (Supplementary Fig. 4e,f).

**Figure 3:**
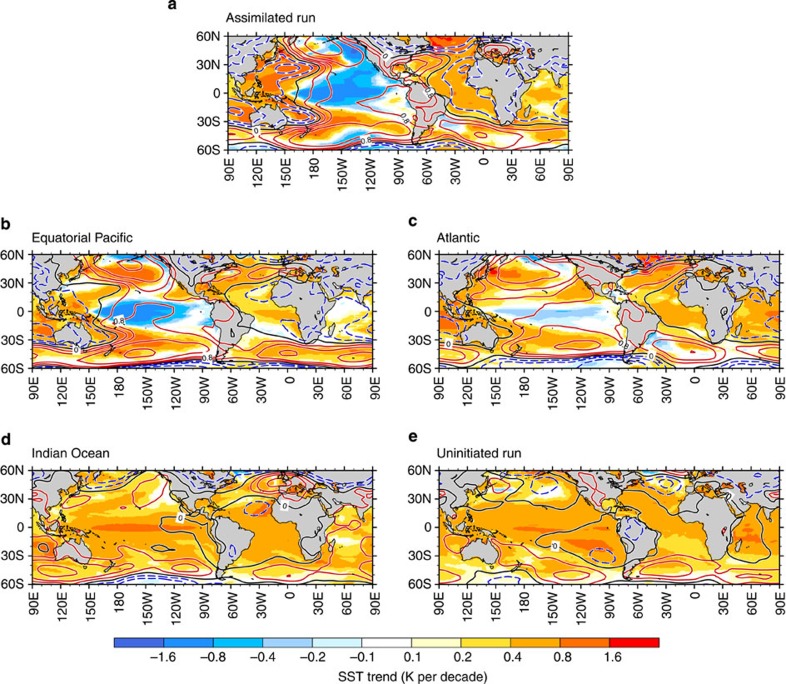
Sea surface temperature and sea-level pressure trends for the 1992–2000 period. Linear trends of SST (shaded) and SLP (contoured) for the 1992–2000 period are obtained in (**a**) the assimilated run, the partial assimilations (**b**) in the equatorial Pacific, (**c**) Atlantic, (**d**) Indian Ocean and (**e**) the uninitialized externally forced twentieth century run. Contour intervals of SLP are 0, ±0.4, ±0.8, ±1.6 and ±3.2 hPa per decade. Red, blue and black lines denote positive, negative and zero contours, respectively.

Our analysis also confirms an active role of the Atlantic Ocean in the recent pause of global warming trend known as the hiatus event^3^,^5^,^12^,^31^. From 1992 to 2000, the first principal components associated with the TBV show a phase change from negative to positive (that is, Atlantic warming and eastern Pacific cooling in Fig. 2e), which contributes to the hiatus event after 2000 as simulated by our forecast (black circle and bars in Fig. 2e). In particular, the eastern tropical Pacific cooling trend offsets some of the greenhouse-induced Atlantic and Indian Ocean warming, thus leading to a weak global SST warming trend. Consistent with this phase change, linear trends of SST and SLP for this period show the key TBV features: trans-basin SST and SLP gradients between the Atlantic–Indian Ocean and the Pacific, La Niña-like SST pattern, and SLP increases in the North and South Pacific Oceans (Fig. 3a). Although our partial assimilation experiments indicate that the equatorial Pacific ocean variability is the major driver for those trends (Fig. 3b) as shown by previous studies^3^,^12^, contributions from the Atlantic Ocean variability explain part of the sea surface cooling in the tropical Pacific and half of the SLP increases in the North and South Pacific Oceans (Fig. 3c). This result further supports our finding that the multi-year predictability in the tropical Pacific partly relies on the long-term memory provided by the Atlantic coupled atmosphere/ocean system. The result is also consistent with a previous study suggesting that the Atlantic SST warming contributed to improve the predictive skills of a La Niña-like SST pattern in the tropical Pacific during the late 1990s climate shift^24^.

### Predictability of the tropical trans-basin variability

**Figure 4:**
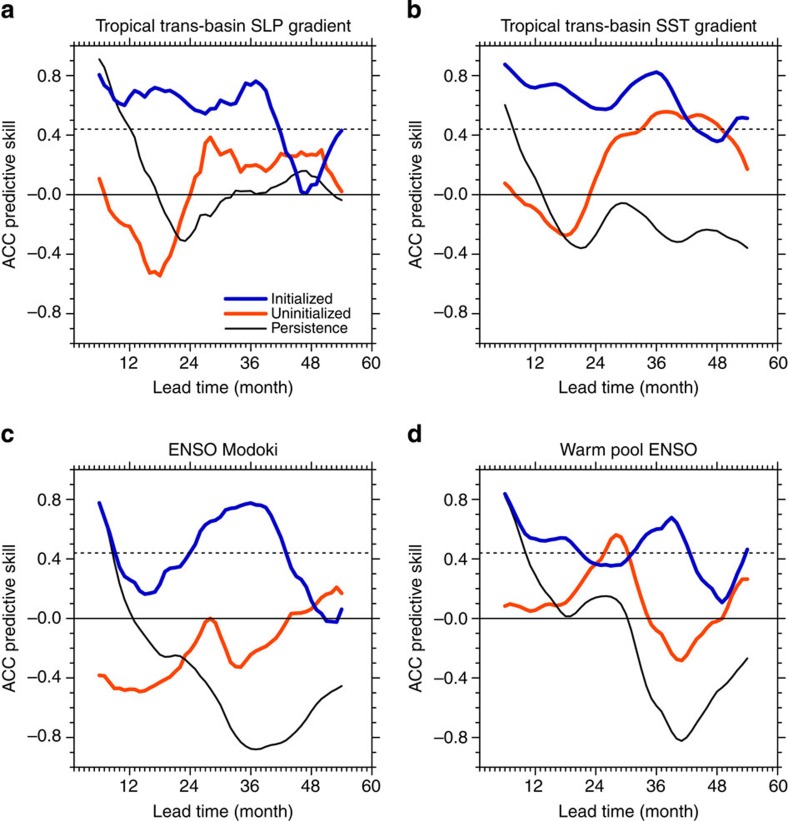
Predictive skills of the tropical indices. Potential predictive skills are obtained from the tropical trans-basin (**a**) SLP and (**b**) SST gradients, (**c**) ENSO Modoki and (**d**) Warm pool ENSO indices in the initialized run (blue), the uninitialized run (red) and persistence (black), measured by anomaly correlation coefficient. A 12-month running mean is applied to indices. Black dotted line denotes the statistical significance at the 90% level with eight degrees of freedom on the basis of one-side Student's *t*-test.

Although ENSO-related SST prediction skill in our MIROC forecasting system quickly decreases at lead times of >1 year (Supplementary Fig. 6), in agreement with previous studies^32^, the tropical TBV indices defined by the trans-basin SLP and SST gradients (see Method) show predictive skill even for lead times of up to 3 years in advance (Fig. 2e and Fig. 4, and Supplementary Fig. 7). This high persistence in TBV compared with ENSO is further illustrated by spectral analysis: ENSO exhibits much less low-frequency variability and a shorter damping timescales compared with the TBV (Supplementary Fig. 8). Because of the larger spatial scales, the TBV averages out many of the higher-frequency sub-basin-scale features, thus enhancing the low-frequency variance in contrast to ENSO. Our partial assimilation experiments also support the trans-basin climate feedbacks (Supplementary Fig. 9) that are associated with an anomalous global Walker Circulation.

The multi-year predictive skill of the TBV mainly relies on the natural variability rather than on the external forcing components. This is illustrated by high prediction skill of the initialized run compared with the uninitialized run (Fig. 4 and Supplementary Fig. 7). However, predictive skill in the uninitialized runs sometimes even outperforms that of the initialized run: the SLP root-mean-squared error skill at 25–29 months (Supplementary Fig. 7a) and the SST skills at 42–49 months lead time (Fig. 4b and Supplementary Fig. 7b). Although the GHG increases contribute to the zonally uniform SST warming, some volcanic events and local aerosol forcings may modulate the trans-basin gradients owing to their zonally asymmetric structures. In fact, the external radiative forcing is the major contributor to SST predictive skill in the Atlantic and Indian Oceans (Fig. 1d–f). Although more accurate estimation of predictive skills would be achieved by the multi-model ensemble in the CMIP5 decadal prediction experiment or the enhanced intervals of the hindcast experiment (for example, initialized every year), our successful prediction at the multi-year lead time provides a new evidence for the importance of the long-term ocean memory in predicting the tropical low-frequency climate variability.

## Discussion

Our results suggest that the Atlantic Ocean plays an important role in low-frequency variability in the tropical Pacific via the atmospheric trans-basin coupling. Although the tropical Pacific variability is still a major driver to induce interannual-to-decadal climate variability in the other basins, the atmosphere–ocean variability in the Atlantic and Indian Oceans can feed back to the Pacific through trans-basin interactions and global displacements of the Walker Circulation. As a result, the tropical TBV predictability exceeds ENSO's predictive skill by a factor of ∼3 (Fig. 4 and Supplementary Fig. 6), despite the fact that our results may be contaminated by model biases and the simple initialization technique.

In the tropical Pacific, the TBV-related SST anomaly pattern also projects onto the El Niño Modoki^33^/warm pool ENSO^34^ mode and onto the Interdecadal Pacific Oscillation^35^,^36^. The simulated 1–12 months predictive skill for the Modoki/warm pool ENSO indices (Fig. 4c,d) is comparable to that of the Niño 3.4 ENSO SST index (Supplementary Fig. 6). This finding is in agreement with previous studies^13^,^19^,^37^,^38^. However, our analysis reveals a considerable recovery of skill for the central Pacific ENSO indices after the 2 or 3 years lead time (Fig. 4c,d), consistent with the idea of a remote impact from the other ocean basins. This suggests that the Modoki/warm pool ENSO mode is not solely an internal Pacific mode, but is partly controlled by Atlantic and Indian Ocean SST anomalies and related displacements of the global Walker Circulation. This conclusion is further supported by the similarity and high correlation (higher than 0.55; above statistical significance at 99.9% level) between the TBV and the Modoki/warm pool ENSO indices (Supplementary Fig. 10). The idea of predictable tropical TBV presented here provides a novel perspective for understanding global-scale climate variability and ENSO flavours. In view of the global impacts of the TBV on precipitation and sea-level anomalies (Fig. 2), operational predictions of the TBV may translate into better assessments of risks in sectors, such as coastal and water management, forestry and agriculture.

## Methods

### Model

The coupled atmosphere–ocean general circulation model adopted here is version 3.2 of the Model for Interdisciplinary Research on Climate (MIROC)^21^ with a T42 spectral resolution and 20 levels on a vertical σ-coordinate. The resolution of the ocean component is 1.4° in longitude and 0.56–1.4° in latitude with 44 vertical levels. No flux correction is applied to exchange heat, water and momentum fluxes between the atmosphere and the ocean. In all the experiments, we use historical observed data of natural and anthropogenic forcings (greenhouse gas and aerosol concentrations, solar cycle variations and major volcanic eruptions) before 2000 and the IPCC A1B-type emissions scenario after 2000.

### Hindcast experiments

We conducted three main experiments using MIROC: the uninitialized, the assimilated and the initialized runs. In the uninitialized run, we prescribed natural and anthropogenic radiative forcings in the coupled model for the period of 1850–2100 with 10 ensemble members starting from different initial conditions obtained from the pre-industrial control simulation. For the 10-member assimilation runs, the model climatology defined by the 1961–1990 period from the uninitialized run was added onto the observed three dimensional oceanic temperature and salinity anomalies from surface to 700-m depth during 1945–2009 (ref. 39). The anomalies outside the sea–ice regions are assimilated into the coupled climate model using the incremental analysis update scheme^40^,^41^. Analysis increments estimated from a temporally and spatially invariant model-to-observation ratio in analysis errors (about one sixth) are added as forcing terms into the model's temperature and salinity tendency equation during an analysis interval of 1 day^16^,^42^. Due to the tight coupling between SLP and SST fields in the tropics, our assimilated run can realistically reproduce the observed trans-basin SLP variability (Supplementary Figs 3, 11 and 12), even without atmospheric assimilation. On the basis of the atmospheric and oceanic initial conditions generated by the 10-member assimilation experiments, we conducted 10-year-long ensemble hindcast experiments initialized from 1 January 1960, 1965, 1970, 1975, 1980, 1985 1990, 1995, 2000 and 2005 (the initialized runs). No post processing for removing an artificial drift is applied to our initialized run because of a negligible climate drift in the tropics during the prediction period. Details of the model experiment and assimilation procedure are described in previous studies^16^,^18^.

### Partial assimilation experiments

We have partially assimilated the observed ocean temperature and salinity anomalies in either the equatorial Pacific (10° S–10° N), the Atlantic (50° S–60° N) and Indian Ocean (30° S–30° N). These partial assimilation experiments consist of 10-member ensembles each and cover the period from 1945 to 2009. The partial assimilation experiments can capture not only the atmospheric response but also the resulting atmosphere–ocean interactions in the other ocean basins, which are allowed to vary freely in response to the atmospheric changes caused by the SST anomalies in the assimilated basins.

### Tropical TBV and ENSO indices

To capture the global-scale characteristics of the TBV, the principal component time series of the 36-month running mean leading EOF mode of SLP is a suitable index. Here, for simplicity and to highlight the important role of tropics, we use the tropical TBV indices on the basis of the zonal trans-basin gradients of SLP and SST (see boxes in Fig. 2a). These surrogate indices show high correlations with the principal components of the leading EOF (Fig. 2e and Supplementary Fig. 2e). The tropical TBV indices on the basis of SLP and SST are defined as the zonal gradient of SLP and SST anomalies averaged and standardized in each region between the tropical central Pacific (15° S–15° N, 180° W–150° W) and the tropical Atlantic–Indian Ocean (15° S–15° N, 40° W–60° E). Note that these indices are more focused on the tropics compared with the global-scale TBV index introduced in a recent study^5^.

The ENSO indices are defined as the zonal gradient of standardized SLP anomalies between the eastern equatorial Pacific (5° S–5° N, 80° W–130° W) and the Indonesia region (5° S–5° N, 90° E–140° E) and the SST anomalies in the Niño 3.4 region (5° S–5° N, 120° W–170° W), respectively. The ENSO index based on the SLP gradient is identical to the equatorial Southern Oscillation index.

## Author contributions

Y.C., M.K., J.-J.L., A.T. and F.-F.J. designed the research. Y.C., T.M. and M.I. performed the experiments. Y.C., M.K., S.-P.X., J.-J.L. and A.T. wrote the paper. All the authors discussed the results and commented on the manuscript.

## Additional information

**How to cite this article:** Chikamoto, Y. *et al*. Skilful multi-year predictions of tropical trans-basin climate variability. *Nat. Commun.* 6:6869 doi: 10.1038/ncomms7869 (2015).

## Supplementary Material

Supplementary Information Supplementary Figures 1-12 and Supplementary References
