# Supplementary material for: Skilful multi-year predictions of tropical trans-basin climate variability
Source: Nat Commun. 2015 Apr 21;6:6869. doi: 10.1038/ncomms7869 (PMC4410635; doi:10.1038/ncomms7869)
Supplement: Supplementary Information — Supplementary Figures 1-12 and Supplementary References [file ncomms7869-s1.pdf]

Supplementary Figures:

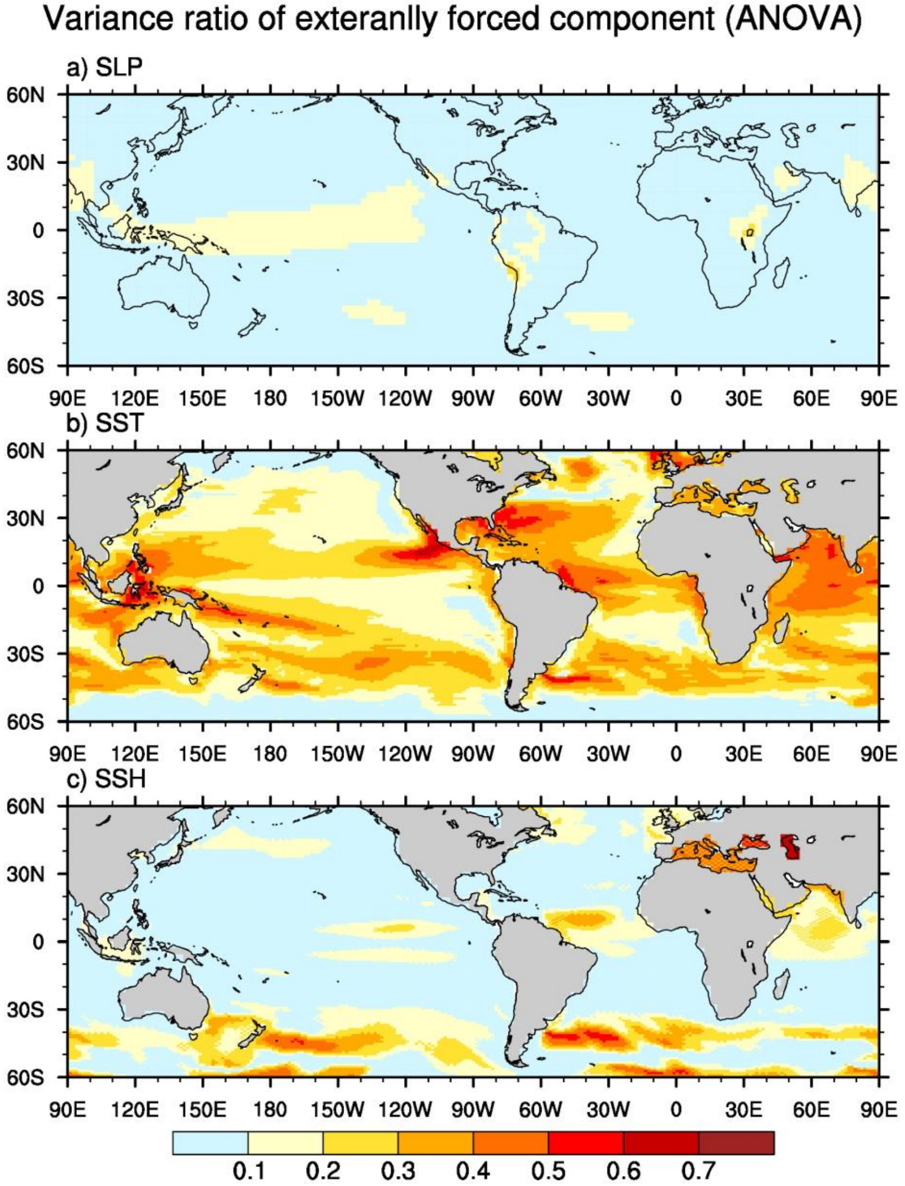

Supplementary Fig. 1: **Contributions from internally generated variability and externally forced component to the total variance.** Variance ratio of externally forced component in annual mean **a**) SLP, **b**) SST, and **c**) SSH, estimated from analysis of variance (ANOVA) technique[1]. In ANOVA, we obtain the signal-to-noise ratio from the ensemble mean and spread for 10 members of the uninitialized run.

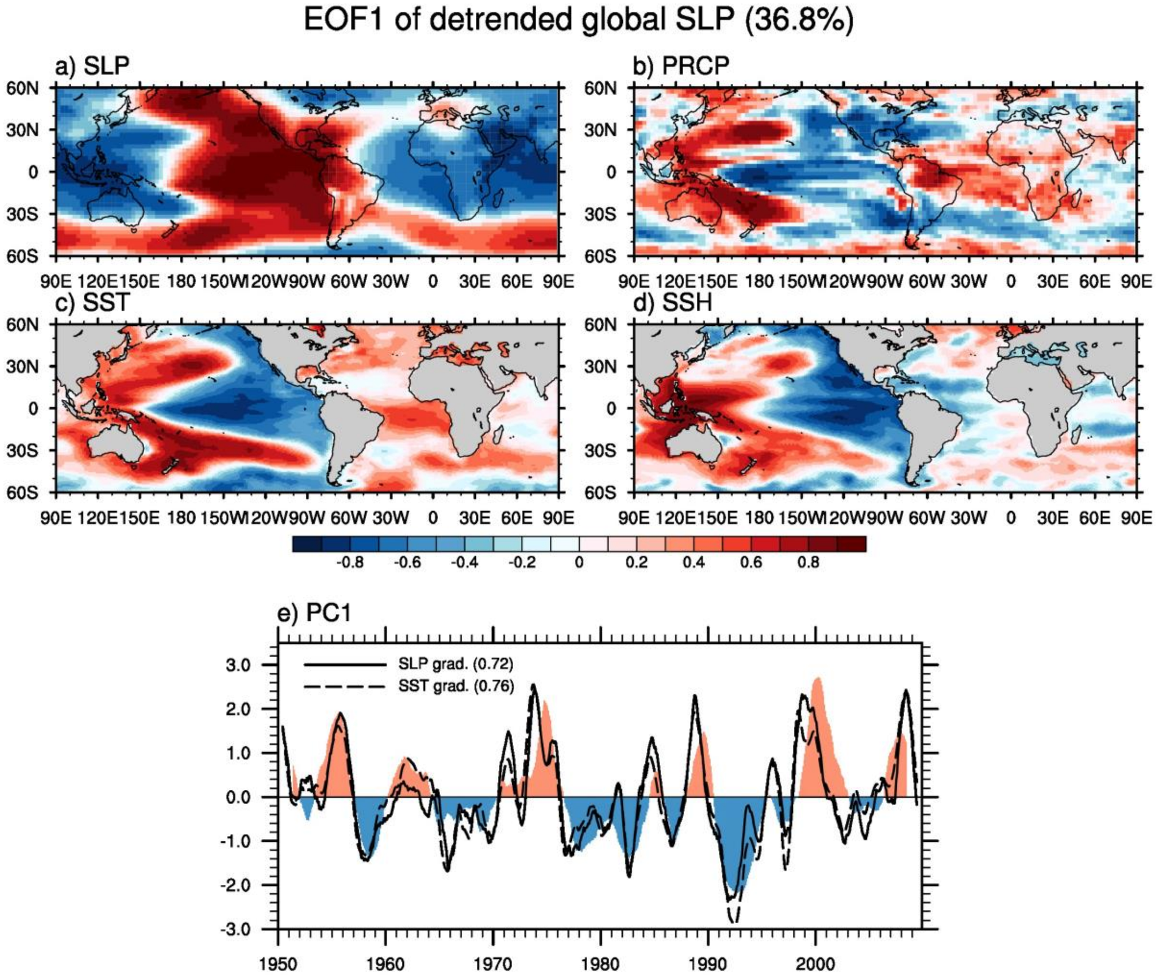

Supplementary Fig. 2: Detrended patterns associated with the Trans-Basin Variability. Correlation maps of **a**) SLP, **b**) precipitation, **c**) SST, and **d**) SSH anomalies with the principal component (shaded in **e**) of the leading correlation-matrix based EOF for global SLP anomalies (60°S-60°N) smoothed by 36-month running mean filter with linearly detrended (explaining 36.8% of variance). Black solid and dashed lines **e** denote the tropical SLP- and SST-based TBV indices with a 12-month running mean filtering (bracket; correlation coefficient with the principal component).

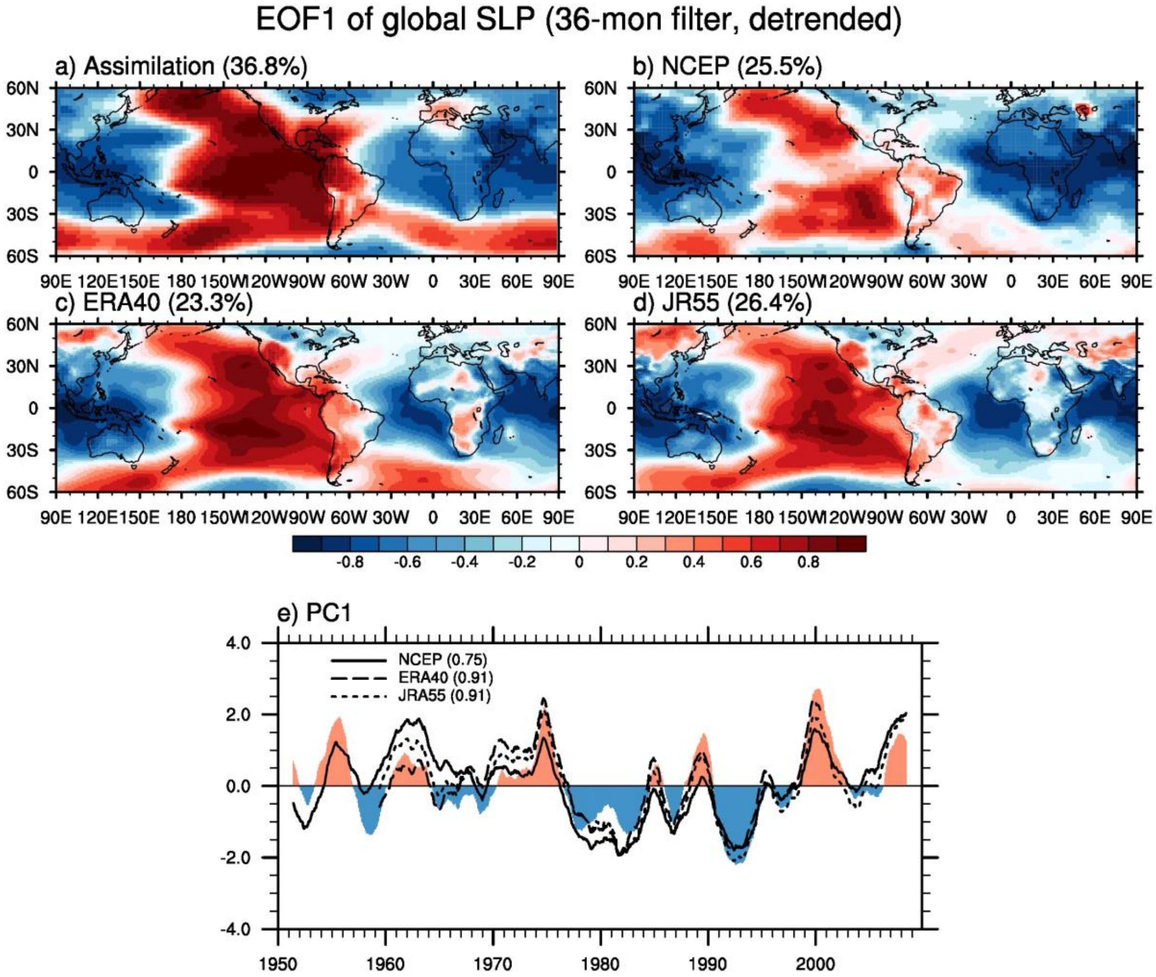

Supplementary Fig. 3: **Observed sea level pressure patterns associated with the Trans-Basin Variability.** First EOF mode of the detrended SLP anomalies (60°S-60°N) smoothed by 36-month running mean filter in **a**) the assimilated run and reanalysis products of **b**) NCEP[2] for 1950-2009, **c**) ERA40[3] for 1958-2001, and **d**) the Japanese 55-year Reanalysis (JRA55)[4] for 1958-2009. Color bar, solid, broken, and dashed lines in **e**) represent PC1 in the assimilated run, NCEP, ERA40, and JRA55, respectively. Their correlation coefficients with the assimilated run are denoted in brackets of legend.

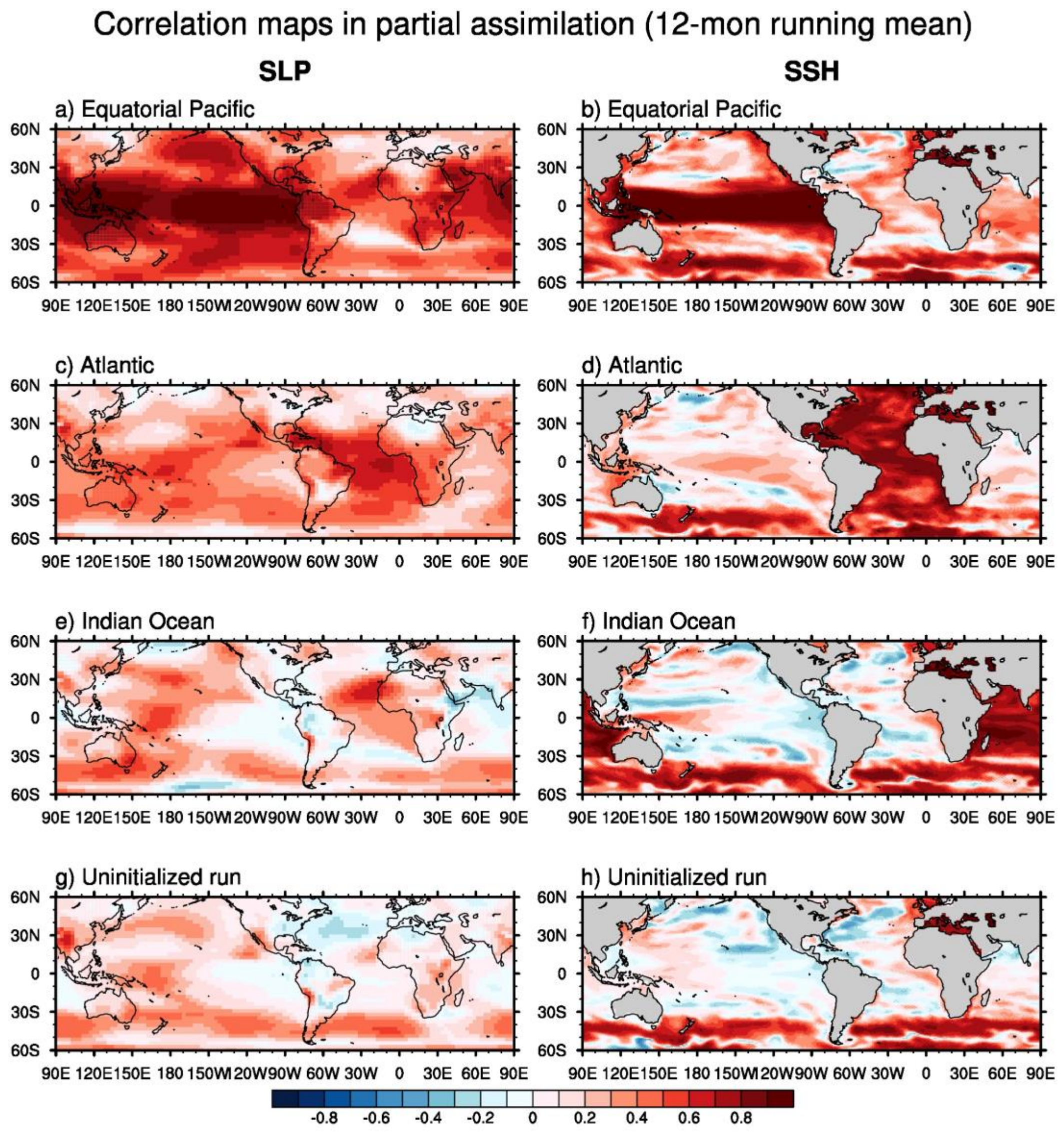

Supplementary Fig. 4: **Remote impact of each ocean basin.** Correlation maps of SLP (left) and SSH (right) anomalies in the assimilated run and with partial assimilation experiments in **a, b**) the equatorial Pacific, **c, d**) the Atlantic and **e, f**) the Indian Ocean, and **g, h**) the uninitialized run. A 12-month running mean filter is applied to all anomalies. Due to a weak local air-sea coupling between SST and SLP anomalies (Supplementary Figure 12), correlation of SLP anomalies in the assimilation run with the partial assimilation of Indian Ocean is unclear, particularly over the tropical Indian Ocean in **e**).

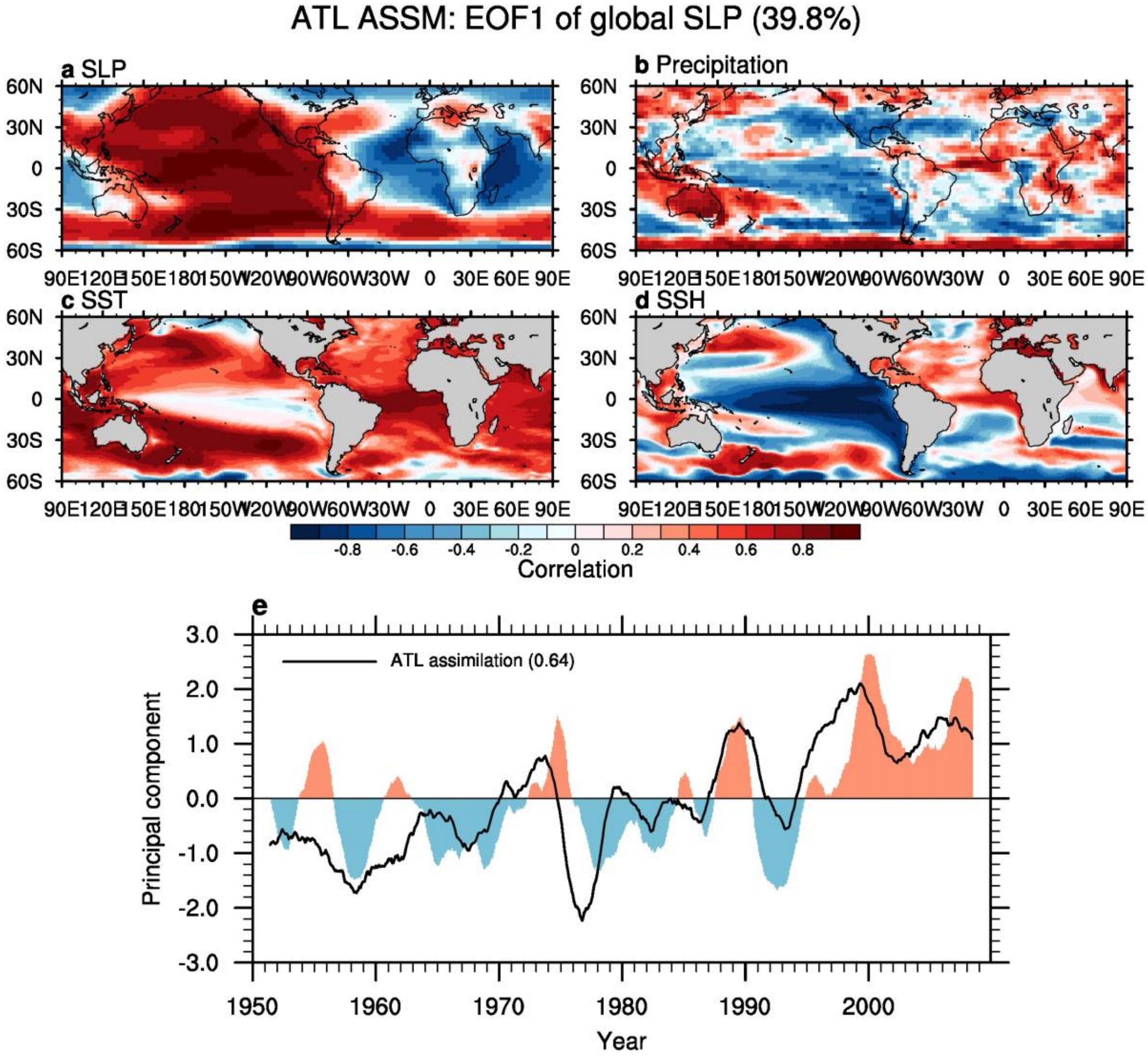

Supplementary Fig. 5: Patterns associated with the Trans-Basin Variability originated from the Atlantic Ocean. Correlation maps of **a**) SLP, **b**) precipitation, **c**) SST, and **d**) SSH anomalies with the principal component (shaded in **e**) of the leading correlation-matrix based EOF for global SLP anomalies (60°S-60°N) smoothed by 36-month running mean filter in the Atlantic partial assimilation experiment. Color and black line **e** denote the principal component in the assimilation run for the global oceans (Same as Fig. 2e) and the Atlantic Ocean (bracket; correlation coefficient with the principal component), respectively.

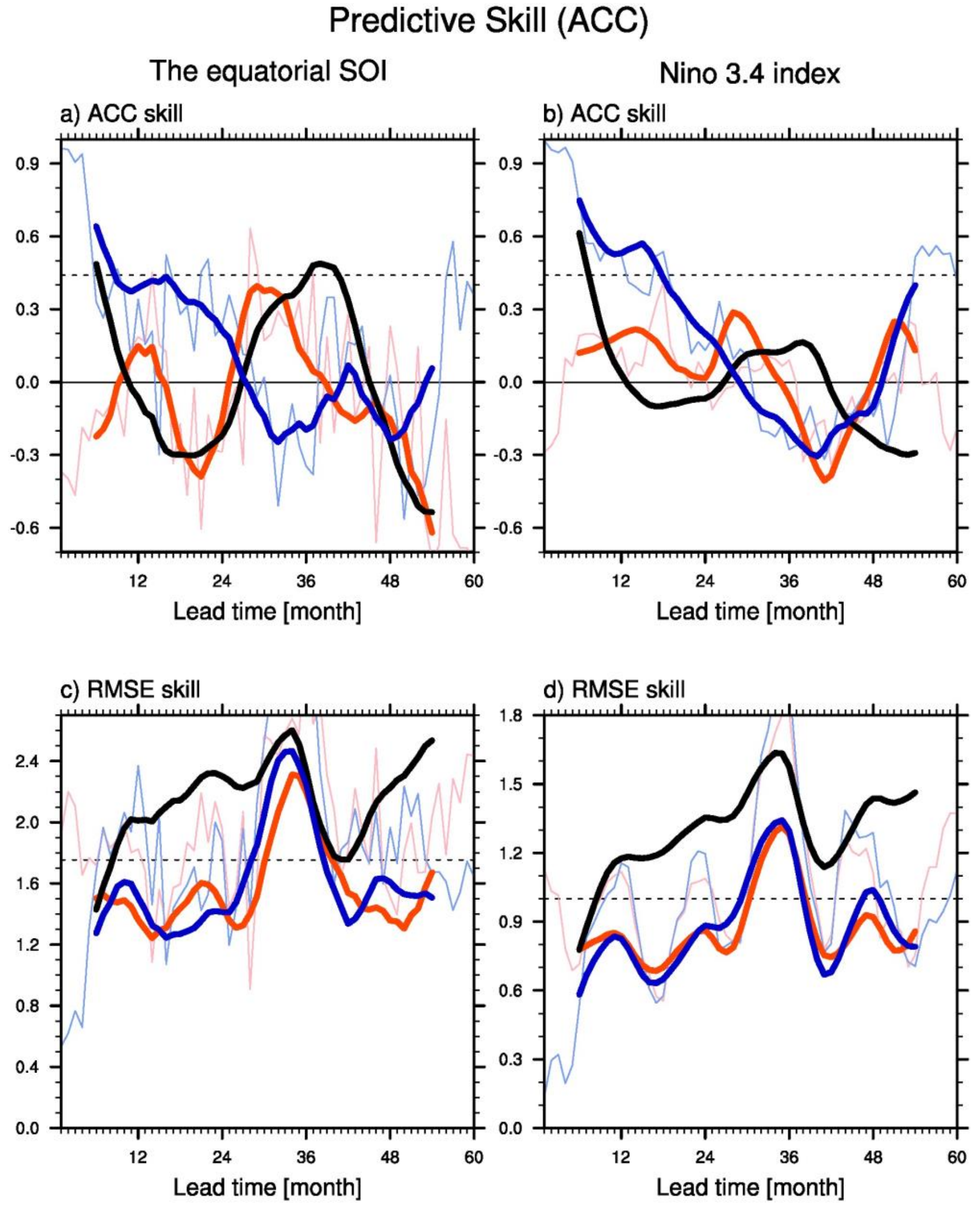

Supplementary Fig. 6: **El Niño Southern Oscillation predictive skills.** Predictive skills of ENSO indices based on **a, c**) the equatorial SOI and **b, c)** Niño 3.4 index in the initialized run (blue), the uninitialized run (red), and persistence (black), measured by anomaly correlation (top) and root-mean-squared error (bottom). Thin and tick lines are predictive skill based on monthly and 12-month running mean indices, respectively. Black dotted line denotes the statistical significance at the 90% level (top panels) and one standard deviation of indices in the assimilation experiment (bottom panels).

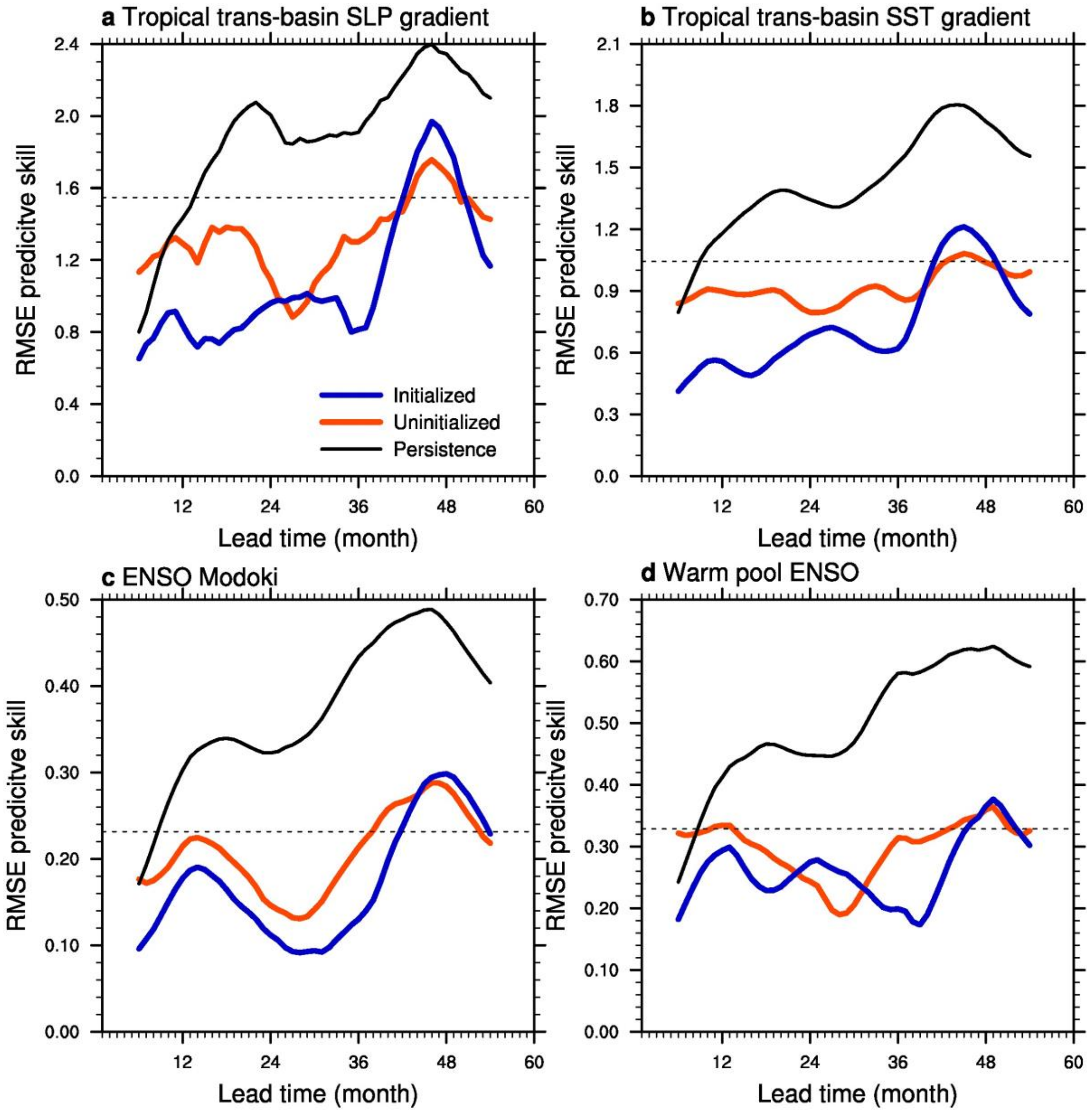

Supplementary Fig. 7: Predictive skills of the tropical indices. Predictive skills of the tropical trans-basin **a**) SLP and **b**) SST gradients, **c)** ENSO Modoki indices[5], and **d**) the warm pool ENSO[6] in the initialized run (blue), the uninitialized run (red), and persistence (black), measured by root-mean-squared error. A 12-month running mean is applied to indices. Black dotted line denotes one standard deviation of indices in the assimilation experiment.

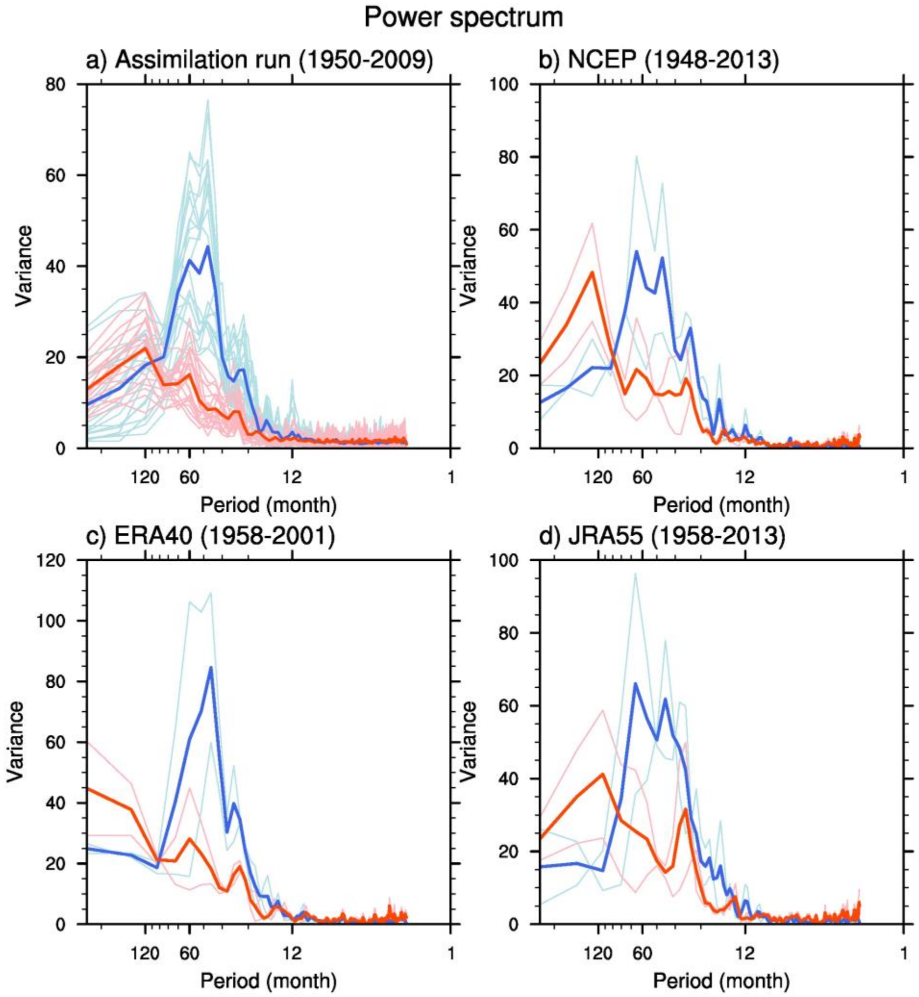

Supplementary Fig. 8: **Power spectrum of Trans-Basin Variability and El Niño Southern Oscillation.** Bartlett power spectrum estimation of the tropical TBV (red) and ENSO (blue) indices in **a**) the assimilated run for the period 1950-2009, **b**) NCEP for 1948-2013, **c**) ERA40 for 1958-2001, and **d**) JRA55 for 1958-2013. Power spectrum is obtained from the monthly basis and linear trends are removed. Light color shows power spectrum of each chunk for windows of 30, 32, 25, 28 years in the assimilated run, NCEP, ERA40, and JRA55. Two chunks in ERA40 have an overlapping period for 3 years. The spectral peak around 4 years in ENSO is much reduced in the tropical TBV index while the tropical TBV index has much power in lower frequency compared to ENSO.

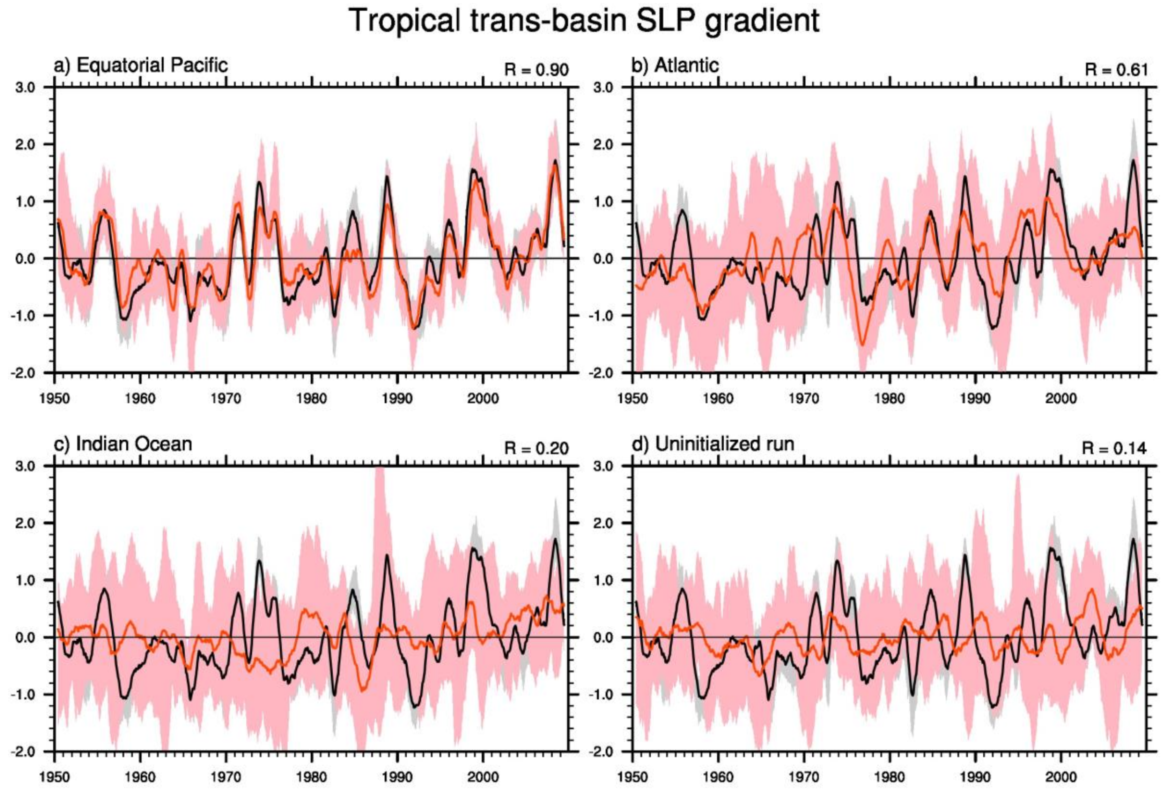

Supplementary Fig. 9: Impact of each ocean basin on the Trans-Basin Variability. The tropical TBV index simulated by the partial assimilation experiments in **a**) the equatorial Pacific, **b**) the Atlantic and **c**) the Indian Ocean, and **d**) the uninitialized run. Solid line and shaded are the ensemble mean and the maximum-minimum range of ensemble members, respectively, in the assimilated run (black) and the partial assimilation experiments (red). Correlation coefficient between black and red lines is denoted in the upper-right corner.

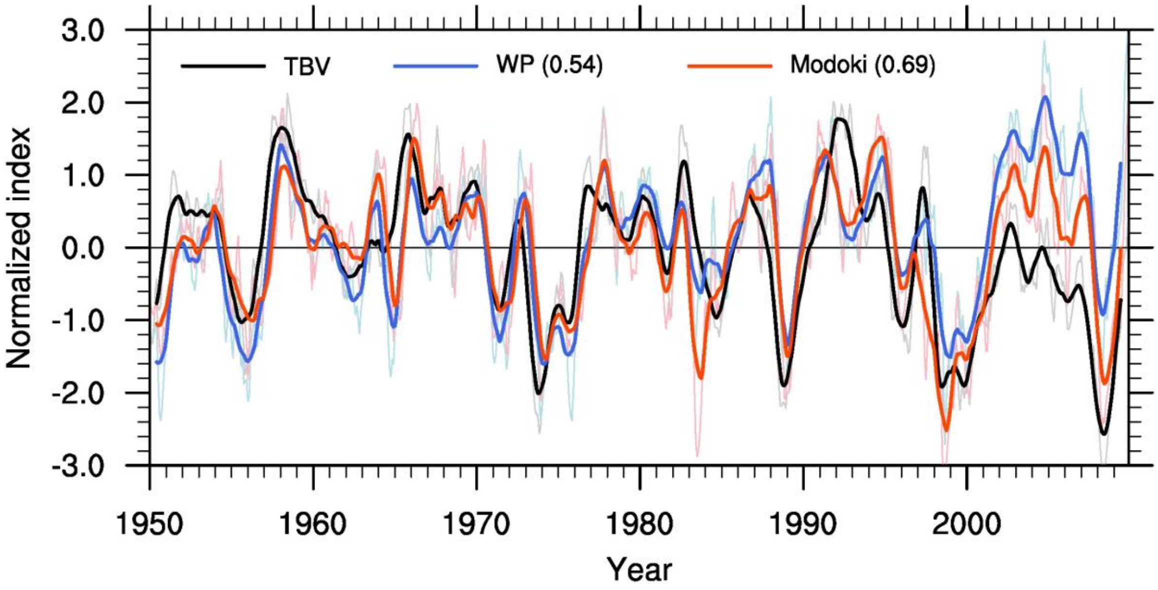

Supplementary Fig. 10: **Indices of Tran-Basin Variability, El Niño Modoki, and warm pool El Niño.** The standardized time series of the SST based TBV (black; sign-reversed), the Modoki (red), and the Warm pool ENSO (blue) indices in the assimilation run. Thin and thick lines are based on monthly and 12-month running mean indices, respectively. Correlation coefficients with the TBV index are denoted in blankets.

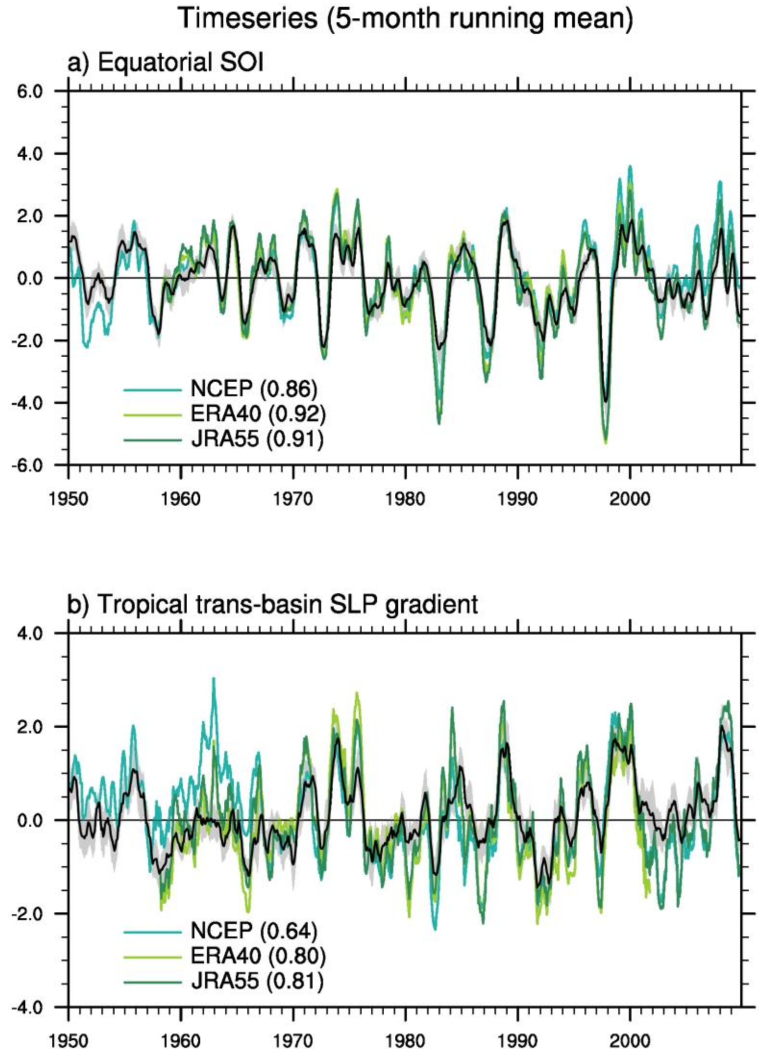

Supplementary Fig. 11: **The El Niño Southern Oscillation and Trans-Basin Variability indices in the reanalysis datasets and the assimilation run.** Time series of **a**) the equatorial SOI and **b**) the tropical trans-basin SLP gradient in the assimilated run and observations. Black line and shaded show the ensemble mean and spread for 10-member ensemble in the assimilated run. Dark, light, and normal greens are observations obtained from JRA55, ERA40, and NCEP reanalysis datasets, respectively. A 5-month running mean filter is applied to time series. Correlation coefficients between observation and assimilation are denoted in blankets.

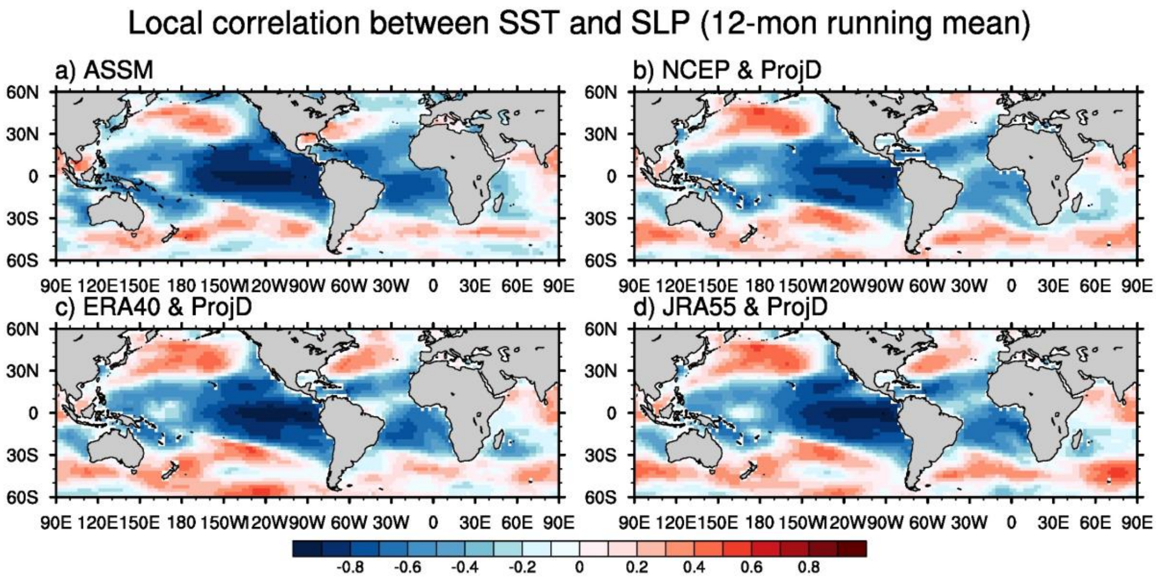

Supplementary Fig. 12: **Local air-sea coupling.** Local correlation coefficient between SST and SLP anomalies in **a**) the assimilation run, **b**) NCEP, **c**) ERA40, and **d**) JRA55 reanalysis datasets for the 1955-2009 period. The SST anomalies in **b-d**) are obtain from the ocean analysis dataset[7] to compare with atmospheric reanalysis datasets. Anomalies are linearly detrended and applied by a 12-month running mean filter.

Supplementary References

1. Rowell, D. P., Folland, C. K., Maskell, K. & Ward, M. N. Variability of summer rainfall over tropical north Africa (1906-92): Observations and modelling. *Quart. J. Roy. Meteorol. Soc.* **121**, 669-704 (1995).
2. Kalnay, E. *et al.* The NCEP/NCAR 40-year reanalysis project. *Bull. Amer. Meteorol. Soc.* **77**, 437-471 (1996).
3. Uppala, S. M. *et al.* The ERA-40 re-analysis. *Quarterly Journal of the Royal Meteorological Society* **131**, 2961-3012, doi:10.1256/Qj.04.176 (2005).
4. Ebita, A. *et al.* The Japanese 55-year Reanalysis "JRA-55" An Interim Report. *SOLA* **7**, 149-152, doi:10.2151/sola.2011-038 (2011).
5. Ashok, K., Behera, S. K., Rao, S. A., Weng, H. & Yamagata, T. El Niño Modoki and its possible teleconnection. *Journal of Geophysical Research: Oceans* **112** (2007).
6. Ren, H. L. & Jin, F. F. Niño indices for two types of ENSO. *Geophysical Research Letters* **38**, doi:10.1029/2010gl046031 (2011).
7. Ishii, M. & Kimoto, M. Reevaluation of historical ocean heat content variations with time-varying XBT and MBT depth bias corrections. *J. Oceanogr.* **65**, 287-299 (2009).
